# COVID-19 Epidemiology, Immunity, and Vaccine Development in Children: A Review

**DOI:** 10.3390/vaccines10122039

**Published:** 2022-11-29

**Authors:** Jaime Fergie, Mary M. Moran, Alejandro Cane, Shanti Pather, Ӧzlem Türeci, Amit Srivastava

**Affiliations:** 1 Driscoll Children’s Hospital, Corpus Christi, TX 78411, USA; 2 Pfizer Inc., Collegeville, PA 19426, USA; 3 BioNTech, D-55131 Mainz, Germany; 4 Pfizer Inc., Cambridge, MA 02139, USA

**Keywords:** COVID-19, SARS-CoV-2, pediatrics, children, vaccine, vaccination

## Abstract

Although pediatric populations experienced lower COVID-19 severity and mortality than adults, the epidemiology of this disease continues to evolve. COVID-19 clinical manifestations in pediatrics commonly include fever and cough, but may differ from adults and by variant. Serious complications, including MIS-C, rarely occur. Although early data showed a decreased likelihood of COVID-19 transmission from children versus adults, outbreaks and viral shedding studies support pediatric transmission potential. Children may mount more robust initial immune responses to SARS-CoV-2 versus adults. COVID-19 vaccines with available pediatric data include BNT162b2, mRNA-1273, CoronaVac, and BBIBP-CorV. Depending on age group and jurisdiction, BNT162b2 and mRNA-1273 have received full approval or emergency/conditional authorization in the United States and European Union from 6 months of age. Clinical trials have shown BNT162b2 and mRNA-1273 safety and high efficacy in pediatric populations, with demonstrably noninferior immune responses versus young adults. Real-world studies further support BNT162b2 safety and effectiveness against the Delta variant. mRNA vaccination benefits are considered to outweigh risks, including myocarditis; however, pediatric vaccination rates remain relatively low. Given a growing body of clinical trial and real-world data showing vaccine safety and effectiveness, pediatric vaccination should be prioritized as an important strategy to control the pandemic.

## 1. Introduction

The COVID-19 pandemic has resulted in >585 million confirmed cases and >6.4 million attributed deaths worldwide as of 12 August 2022 [1]. Although children and adolescents typically experienced less severe COVID-19 and accounted for fewer deaths than adults, according to a September 2021 report by the World Health Organization (WHO) [2], the pandemic adversely affected this age group through education interruption, detrimental social–emotional development, and mental health effects [3]. Additionally, children and adolescents may experience multisystem inflammatory syndrome (MIS-C) [4,5] and other long-term COVID-19-associated sequelae [6,7]. Analyses of hospitalization rates during the Omicron variant wave revealed a disproportionate number of hospitalizations among children and adolescents, particularly those aged <5 years, compared with other age groups [8,9,10].

**Figure 1:**
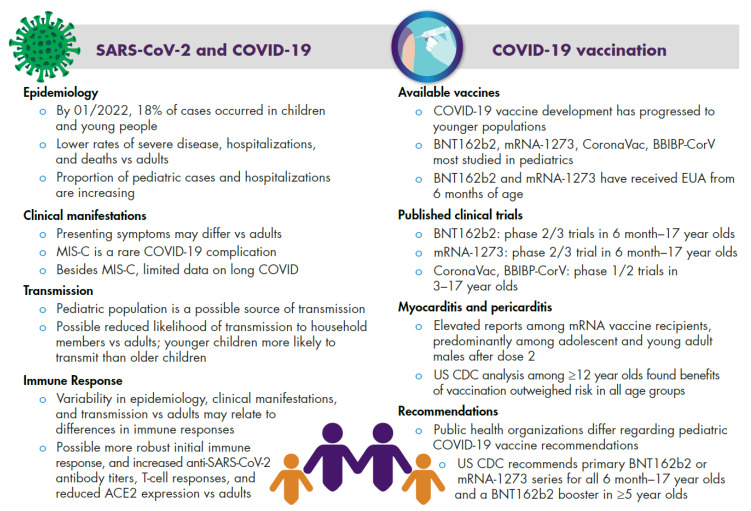
Summary of SARS-CoV-2 and COVID-19 in Children and Adolescents. ACE2, angiotensin-converting enzyme 2; CDC, US Centers for Disease Control and Prevention; EUA, emergency use authorization; MIS-C, multi-inflammatory syndrome in children.

Due to the dynamic nature of COVID-19 and rapidly emerging data, comprehensive evaluation of SARS-CoV-2 infection and COVID-19 vaccination in children and adolescents is beneficial. We reviewed the epidemiology of COVID-19 in children, adolescents, and young people up to 20 years of age, as well as clinical manifestations, transmission, and immune response. Current knowledge of COVID-19 characteristics and vaccination in children and adolescents is summarized in Figure 1 and discussed in more detail below. We also reviewed clinical trial and real-world data for the various available COVID-19 vaccines and vaccination recommendations for pediatric populations. In general, we have focused on evidence from larger studies where possible to enable more reliable and representative inferences among a large and rapidly growing body of disparate research.

## 2. COVID-19 in Children and Adolescents

### 2.1. Epidemiology

As of March 2022, 21% of all COVID-19 cases worldwide have occurred in <20-year-olds [11]. The true burden in this population is likely underestimated, partly because of generally milder symptoms, leading to less testing, compared with adults [2]. Because widespread pediatric vaccination recommendations are lacking, children and adolescents comprise an increasing proportion of the unvaccinated population, thereby constituting a greater proportion of cases associated with emerging variants (e.g., Delta, Omicron) [9,12,13,14,15]. 

Severe disease is uncommon among SARS-CoV-2–infected children (Figure 1). In a May 2020 evaluation of 28 studies, most children with SARS-CoV-2 (n = 1614, predominantly from China) presented with mild (37%) or moderate (45%) COVID-19; 16% were asymptomatic [16]. A systematic review of 65 publications dated January–April 2020 found that 2.6% of cases among ≤18-year-olds (n = 7480; mostly from Italy, China, and the United States) were severe or critically ill [17]. A US study of 3106 children with COVID-19-associated hospitalizations from March 2020 to May 2021 (i.e., spanning multiple variant waves [15]) identified having ≥1 comorbidity (e.g., obesity, chronic lung disease, airway abnormalities, cardiovascular disease, immunocompromising conditions) as a potential risk factor for severe disease in multivariable analyses [18]. 

Although children and adolescents initially comprised a small proportion of overall COVID-19 hospitalizations, those rates have recently increased (Figure 1), particularly among young children, coinciding with the emergence of highly transmissible variants [8,10,15]. In 0–4-year-olds in the United States, COVID-19-associated hospitalization rates remained <3/100,000 through November 2021 but peaked at 16.2/100,000 in January 2022 during the Omicron wave [8,15]. For children <5 years of age, COVID-19 vaccination was recommended, and vaccines only became available in June 2022 [19]; therefore, it is reasonable to infer that the increase in hospitalizations in this age group in January 2022 was caused by a highly transmissible variant, Omicron, circulating among an unvaccinated subpopulation. This inference is supported by CDC analyses of COVID-19 epidemiology during Omicron predominance until June 2022 that showed that children aged 6 months to 4 years old were at risk of severe illness from COVID-19. More than 50% of hospitalized children in this age group had no underlying conditions. COVID-19-associated hospitalization severity among 6-month-olds to 4-year-olds was similar to or greater than older children and adolescents, and the COVID-19 hospitalization burden among 6-month-olds to 4-year-olds was similar to or exceeded that of other pediatric vaccine-preventable diseases [20].

Consistent with low severe disease rates in children and adolescents, this population currently comprises 0.4% of COVID-19-associated deaths globally, 58% of which occur in 10–19-year-olds [11]. A Brazilian nationwide database study of 11,613 hospitalized <20-year-olds with SARS-CoV-2 during February 2020–January 2021 (i.e., largely before any documented variant waves [15]) identified indigenous ethnicity, presence of comorbidities, and age <2 or 12–19 years as significantly associated with increased mortality risk [21]. 

### 2.2. Clinical Manifestations

Common COVID-19 presenting symptoms differ between pediatric and adult populations (Figure 1). Data from systematic reviews showed that children and adolescents with SARS-CoV-2 infection exhibited symptoms consistent with acute respiratory infections, including fever and cough in up to 60% of individuals and high fevers in up to one-third of children [17,22]. Compared with adults, children more commonly presented with extra-respiratory symptoms, particularly diarrhea (9%) and vomiting (7%).

Children with cancer, stable immunosuppression following transplantation, well-controlled HIV infection, or allergic asthma appear to experience COVID-19 infection similarly to healthy children [23]. Pediatric COVID-19 symptoms may vary with emerging variants; in particular, croup rates (i.e., laryngotracheobronchitis or airway obstruction) have markedly increased in connection with the initial Omicron wave [24].

COVID-19-associated complications identified in hospitalized children (n = 671) in an international cohort study (February–October 2020) included laboratory abnormalities (e.g., elevated C-reactive protein, ferritin, procalcitonin) and complications such as cardiac arrhythmias (15% overall), viral pneumonia (13%), and respiratory failure (11%) [25]. Other serious but rare adverse events (AEs) observed after SARS-CoV-2 infection include acute kidney injury, deep-vein thrombosis, myocardial infarction, myocarditis/pericarditis, and pulmonary embolism [26]. 

The rare COVID-19-associated complication MIS-C can develop weeks after infection [4,5] (Figure 1). Approximately two-thirds of 570 MIS-C cases reported in the United States during March–July 2020 occurred among children without underlying medical conditions [27]. An MIS-C case is defined as a <21-year-old patient who has current/recent SARS-CoV-2 infection and presents with fever, inflammation, and clinically severe illness necessitating hospitalization and involving ≥2 organs [5]. Common presentations include persistent fever, abdominal pain, vomiting, diarrhea, skin rash, and mucocutaneous lesions. In severe cases, patients may experience hypotension and shock; some patients may develop myocarditis, cardiac dysfunction, and acute renal damage. MIS-C treatment is limited to supportive strategies and treatment of underlying inflammatory processes. As of 1 August 2022, 8798 MIS-C cases and 71 associated deaths have been reported in the United States; median patient age was 9 years, and 57% were Hispanic/Latinx or Black, and 61% were male [4].

Despite increasing evidence of chronic symptoms in adults diagnosed with acute COVID-19, data on pediatric long COVID are comparatively scarce and vary widely (Figure 1) [6,7]. In a cross-sectional study in Italy (March–October 2020), long COVID symptoms developed in 43% of ≤18-year-olds (n = 129) >60 days after microbiologically confirmed COVID-19 [6]. Symptoms included insomnia, nasal congestion, fatigue, myalgia, concentration difficulties, and joint pain (7–19% each). A UK cohort study found 66.5% and 53.3% of 11–17-year-olds who tested positive (n = 3065) or negative (n = 3739) for SARS-CoV-2 during January–March 2021 reported symptoms 3 months later; 30.3% and 16.2%, respectively, reported ≥3 symptoms [7].

### 2.3. Transmission

SARS-CoV-2 transmission appears to be influenced by a variety of factors in the infected person, such as presence and type of symptoms, nature and duration of exposure, variant, and viral load [28]. Studies from 2020 with ancestral SARS-CoV-2 and before the identification of variants of concern have shown that viral load dynamics can differ between children and adults. A Swiss study (n = 8027) identified a modest positive correlation between age and viral load at diagnosis; pediatric age groups exhibited steeper increases in nasopharynx (NP) viral load the first day after symptom onset versus adults, but viral shedding patterns appeared similar across age groups [29]. A separate analysis (March–June 2020; n = 68) showed that the duration of viral shedding and median time to clearance after infection was significantly longer among 6–15-year-old participants (median, 44 days) compared with 16–22-year-old participants (median, 18 days), whereas 0–5-year-olds (median, 22 days) did not differ significantly from either older age group; in addition, median time to detect neutralizing antibodies did not differ across the three age groups [30]. Viral RNA concentration is a surrogate for the presence of infectious virus and can remain high for many days after culture-competent viral isolation is no longer successful [31]. However, culture-competent SARS-CoV-2 was isolated from 52% (12/23) of NP samples of <16-year-old children infected with SARS-CoV-2 [32]. Taken together, the prolonged presence of virus in the nasopharyngeal passages of children after infection with SARS-CoV-2 suggests that they may have a tenable role in transmission; however, it is unclear whether the persistence of viral genome correlates with transmissibility [30].

Household studies enable a better assessment of transmission. Reviews of studies from the early pandemic phase (published by August–October 2020) indicated that household and cluster index cases were not commonly attributable to children [33,34], although that may be attributed to the closing of in-person learning and extracurricular activities [35]. However, outbreaks occurring in settings frequented by children and adolescents (e.g., sporting events and schools) and other transmission studies have shown that this population can also transmit SARS-CoV-2 (Figure 1) [35]. A review of data published during December 2019–June 2020 indicated that children were less likely than adults to transmit SARS-CoV-2 to household members (8 vs. 205 of 213 clusters) [33]. A Canadian study of 6280 households with a pediatric index COVID-19 case (June–December 2020) indicated a higher likelihood of younger versus older children to transmit SARS-CoV-2 [36]; however, adolescents may have greater overall transmission due to comparatively increased social interaction [35]. SARS-CoV-2 transmission among children and adolescents has likely increased with the predominance of highly transmissible variants [35].

### 2.4. Immune Response

COVID-19 epidemiology and clinical manifestations differ significantly between children and adults, a pattern that has remained consistent across the emerging SARS-CoV-2 variants (Figure S1) [37]. Adult COVID-19 patients exhibit respiratory symptoms and can progressively deteriorate to acute respiratory distress syndrome, whereas children infected with SARS-CoV-2 tend not to exhibit respiratory illness but can suffer from MIS-C, a rare and life-threatening severe COVID-19 complication [38]. Potentially relevant immunologic parameters include comparatively lower levels of reduced accumulated endothelial dysfunction and related comorbidities, lack of immunosenescence associated with reduced viral clearance, reduced angiotensin-converting enzyme-2 (ACE2) expression, and higher lymphocyte and cytotoxic T-cell counts among children (Figure 1) [37,39].

Studies have attempted to characterize the difference in immune mediators between children and adults, although no edifying patterns have emerged thus far. In one US study (November 2020–January 2021) of individuals presenting to the emergency department with SARS-CoV-2 infection, significantly higher levels of cytokines, including interferon-gamma (IFN-γ), IFN-alpha-2 (IFN-α2), interleukin-1-beta (IL-1β), IL-8, and IFN-γ-inducible protein 10, were detected in nasopharyngeal samples collected from children (n = 12) compared with adults (n = 27) [40]. Gene expression levels corresponding to CD4, CD8A, and CD20 were also higher in the pediatric samples, further suggesting a more robust initial cellular immune response among children (Figure 1), but the total immunoglobulin G (IgG) and IgA antibody levels did not differ between age groups. On the other hand, a separate study (March–June 2020; n = 79) found that among adult COVID-19 patients, spike-specific IgG, IgM, and IgA antibody levels increased with disease severity and were significantly higher compared with levels in pediatric patients with MIS-C; antibody levels were similar among children with and without MIS-C [41]. Furthermore, anti-nucleoprotein IgG plasma levels and neutralizing antibodies were significantly lower in children than in adults, regardless of their MIS-C status [41]. Examining SARS-CoV-2–specific immune responses by age group (April–August 2020) found that serum IgG levels negatively correlated with age among ≤18-year-olds (n = 85), but a reverse trend was observed among adults (n = 3648); among ≤24-year-olds (n = 126), neutralizing antibody levels also negatively correlated with age [42].

Studies have also evaluated whether pre-existing immunity to common circulating human coronaviruses (HCoVs) may account for the observed age-related differences in immune responses to SARS-CoV-2 infection. In one study that examined HCoV-reactive antibody cross-reactivity with SARS-CoV-2 spike protein, 302 uninfected adults showed low (5%; n = 16) cross-reactivity compared with children 6–16 years old (62%; n = 43); the authors speculated that these differences reflected comparatively higher pediatric HCoV infection rates and may potentially affect disease severity and transmission patterns of COVID-19 [43].

## 3. Pediatric COVID-19 Vaccine Development

**Figure 2:**
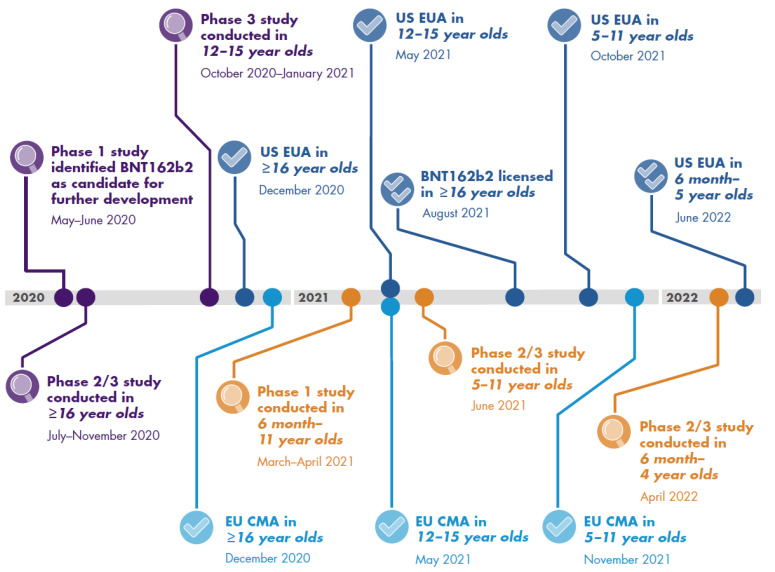
Timeline of the Development of BNT162b2 [46,47,51,52,53,54,55,56,57]. CMA, conditional marketing authorization; EUA, emergency use authorization.

As of 5 July 2022, 168 and 198 vaccines were in clinical or preclinical development, respectively. Thirty-seven COVID-19 vaccines have received licensure or emergency use authorization (EUA) [44,45]. Vaccines were initially tested in adults, and subsequent studies have included progressively younger populations (Figure 1). Vaccines studied in pediatric populations include two mRNA vaccines, BNT162b2 and mRNA-1273, and the inactivated CoronaVac and BBIBP-CorV vaccines (Table 1 [46,47,48,49,50]). BNT162b2 was initially authorized in the United States and in the European Union in ≥16-year-olds as a two-dose 30 µg primary series (Figure 2 [46,47,51,52,53,54,55,56,57]) [54,58], followed by authorization for 12–15-year-olds and a booster dose authorization in the United States for ≥12-year-olds [52,54]. The two-dose 30 µg primary series is now fully licensed in the United States for ≥16-year-olds [54]. For the pediatric age group, a two-dose 10 µg BNT162b2 primary series in 5–11-year-olds and a three-dose 3 µg BNT162b2 primary series in 6-month-olds to 4-year-olds have been authorized in the United States and the European Union, respectively [53,54,57]. A two-dose 100 µg mRNA-1273 series is licensed in the United States for vaccination of ≥18-year-olds [59] and in the European Union for ≥12-year-olds [60]. For the pediatric age group (6–11-year-olds), a two-dose 50 µg series is licensed in the European Union [60]. In the United States, a two-dose mRNA-1273 series for children 6–11 years old (50 µg) and 6 months–5 years old (25 µg) has been authorized [61]. CoronaVac and BBIBP-CorV are in early-stage clinical trials in children [49,50] and have received Emergency Use Listing from the WHO for adult vaccination using a two-dose series [62,63]. 

### 3.1. Pediatric Clinical Trials

#### 3.1.1. Clinical Trial Data on Immunogenicity and Vaccine Efficacy

A phase 3 assessment of a two-dose 30 µg BNT162b2 series in 12–15-year-olds (n = 2260) observed 100% vaccine efficacy (VE) ≥ 7 days after dose 2 (Table 1) [46]. Immune responses to BNT162b2 in 12–15-year-olds were demonstrably noninferior to that of 16–25-year-olds. Among 5–11-year-olds, a two-dose 10 µg BNT162b2 primary series was selected based on safety and immunogenicity findings from a phase 1 dose-level identification study (n = 48) [47]. The phase 2/3 study component in this age group (n = 2268) reported 90.7% VE from 7 days after dose 2 using this dose level; neutralizing titers were noninferior to those in young adults. Among 6-month–4-year-olds, a three-dose 3 µg BNT162b2 primary series was selected for evaluation based on phase 1 dose-level finding results and phase 2/3 immunobridging findings after two doses [65]. In the phase 2/3 assessment of this study (6–23 months old, n = 1776; 2–4 years old, n = 2750), neutralizing titers after three doses in this age group were noninferior to those in young adults after two doses [57].

A phase 2/3 study in 12–17-year-olds (n = 3726) administered a two-dose 100 µg mRNA-1273 series showed noninferiority of neutralizing titers compared with those in young adults (Table 1) [48]. VE estimates were 56% for preventing SARS-CoV-2 infection 14 days after dose 2 and 93% using a less stringent COVID-19 definition. In a phase 2/3 study in 6–11-year-olds (n = 4016) administered a two-dose 50 µg mRNA-1273 series, noninferiority of neutralizing titers compared with those in young adults who received two doses of 100 µg mRNA-1273 was shown, and the estimated VE against COVID-19 occurring ≥14 days after dose 1 was 88% [64]. In the same phase 2/3 study, noninferiority of a two-dose 50 µg mRNA-1273 series in 6-month–5-year-old children compared with young adults was also shown [61].

Two-dose schedules of 1.5 µg and 3.0 µg CoronaVac were investigated in a phase 1/2 randomized trial that included 3–17-year-olds (n = 552; Table 1) [49]. While both doses induced neutralizing antibody titers, higher titers were associated with the 3.0 µg dose, supporting further pediatric evaluations, including in a phase 3 study in ≥6-month-old children (NCT04992260). Three-dose BBIBP-CorV schedules were investigated in a phase 1/2 trial in 3–17-year-olds (n = 1008); the 4 µg dose was selected for further study based on humoral responses [50].

#### 3.1.2. Clinical Trial Safety Data

BNT162b2 safety data in 6-month–15-year-old children are available from clinical studies (Table 1). For both the phase 3 study in 12–15-year-olds and the phase 2/3 study in 5–11-year-olds, injection site pain was the most frequently reported local reaction (71–86% across doses) but was generally mild or moderate in severity and resolved within 1–2 days [46,47]. Systemic events were more frequent after dose 2 compared with after dose 1; these were also generally mild or moderate in severity and most frequently included fatigue or headache (dose 1, 34–60% and 22–55%, respectively; dose 2, 39–66% and 28–65%). Fevers ≥38 °C occurred in 20% of 12–15-year-olds after dose 2 and 8.3% of 5–11-year-olds following either dose; fever >40 °C occurred in a single participant in each age group. Antipyretic use was more frequent after dose 2 (51% vs. 37% after dose 1 among 12–15-year-olds). Few serious AEs (SAEs) were reported through 1 month after dose 2; none were considered vaccine related. No myocarditis/pericarditis cases (discussed later) were observed. Lymphadenopathy was reported in ≤0.9% of BNT162b2 recipients in each study, and no recipients experienced vaccine-related anaphylaxis. For the phase 2/3 study in 6-month–4-year-olds, the most common local reaction after any dose was injection site tenderness/pain (47–68%) and the most common systemic event was irritability (68%) and fatigue (45%) in 6–24-month-olds and 2–4-year-olds, respectively; lymphadenopathy was reported in ≤0.2% of participants [57].

For the mRNA-1273 vaccine, injection site pain was the most commonly reported local reaction (92–93% across doses) in the phase 2/3 adolescent study but was mostly mild or moderate in severity [48]. Headache and fatigue were the most commonly reported systemic events and were mostly mild or moderate in severity (dose 1, 45% and 48%, respectively; dose 2, 70% and 68%); 1.9% of participants reported fever ≥39 °C–40 °C after dose 2, and 1 participant had fever >40 °C. Solicited local reactions and systemic events had a 4-day mean duration. Injection site lymphadenopathy occurred in 4.3% of recipients; none of the few SAEs reported ≤28 days after either dose were considered vaccine related. In the phase 2/3 study in 6–11-year-olds, the most common local reaction was pain (dose 1, 94%; dose 2, 95%) and the most common systemic events were headache (dose 1, 31%; dose 2, 54%) and fatigue (dose 1, 43%; dose 2, 65%); most reactions were grade 1 or 2 and persisted for a median of 2 or 3 days, with a median duration of 1 day for fever [64]. In 6-month–5-year-old children, the most common local reaction was pain (dose 1, 37%; dose 2, 46%) and the most common systemic event was irritability/crying (dose 1, 68%; dose 2, 64%) [61].

The phase 1/2 CoronaVac study reported injection site pain as the most frequent local reaction (16% across both dose levels) and fever as the most frequent systemic event (4–5%) [49]. No SAEs were reported among vaccine recipients. In the phase 1/2 BBIBP-CorV study, injection site pain (7%) and fever (9%) were the most common local and systemic reactions, respectively [50]. 

### 3.2. Real-World Evidence

In contrast to data obtained from traditional clinical trials, real-world evidence (RWE) is clinical evidence regarding the use of a medical product and its associated benefits or risks derived from the analysis of data from heterogenous sources such as electronic health records (EHRs), health insurance claims, product and disease registries, and patient-generated data including in-home use settings; RWE can be developed through different study designs or analyses such as randomized trials, pragmatic trials, and prospective or retrospective observational studies [66].

#### 3.2.1. Real-World Vaccine Effectiveness

Observed BNT162b2 efficacy in adolescents is supported by real-world effectiveness data from Israel during the 2021 Delta outbreak showing 91.5% adjusted vaccine effectiveness against SARS-CoV-2 infection at 2–4 weeks after dose 2 in 12–15-year-olds (n = 8268) [67]. Another concurrent real-world analysis in Israel estimated 90% vaccine effectiveness against infection among vaccinated 12–18-year-olds compared with matched controls (n = 94,354 each) [68]. Similarly, a test-negative real-world analysis of US data during the Delta wave identified 93% BNT162b2 effectiveness following two doses given 3 weeks apart against COVID-19 hospitalization among 12–18-year-olds (n = 464 total) [69]; a separate analysis estimated 91% effectiveness against MIS-C in this age group (n = 102 cases) [70]. In the United Kingdom, which adopted a two-dose BNT162b2 regimen with an extended 8–12-week interval for adolescents, a test-negative study during the Delta wave estimated 75.4–75.9% effectiveness against symptomatic disease among 12–17-year-olds (n = 543,017) after dose 1, which declined to 40–46.8% by 8–9 weeks but rebounded to 94.6% by 2–9 weeks after dose 2, re-emphasizing that the second dose is essential for protection [71].

#### 3.2.2. Real-World Safety

Early real-world safety data for BNT162b2 are now available from the US vaccination program. After administration of 8.9 million doses to 12–17-year-olds, there were 9246 AE reports, of which 90.7% (n = 8383) were nonserious [72]. Corresponding values in 5–11-year-olds were 4249 and 97.6% (n = 4149) [73]. No data in either age cohort suggested a causal association between death reports and vaccination. Overall, the data indicated a BNT162b2 safety profile similar to that observed in preauthorization clinical trials. 

#### 3.2.3. Myocarditis and Pericarditis

A signal of elevated, albeit rare, rates of myocarditis and pericarditis was identified among mRNA COVID-19 vaccine recipients, predominantly adolescent and young adult males after dose 2 (Figure 1) [74]. In the United States, myocarditis incidence rates as of June 2021 as reported to the Vaccine Adverse Event Reporting System (VAERS) were 40.6 and 4.2 cases per million mRNA COVID-19 vaccine second doses among 12–29-year-old males and females, respectively, reported ≤7 days after dose receipt; rates among ≥30-year-olds were 2.4 and 1.0 per million doses, respectively [74]. Electronic medical record data from the Vaccine Safety Datalink supported the association between the second COVID-19 mRNA vaccine dose and myocarditis [74]. A CDC-conducted benefit–risk analysis of mRNA COVID-19 vaccination found that vaccination benefits outweighed myocarditis risk (Figure 1), based on VAERS data, in all age groups assessed (≥12 years) [74]. An updated August 2021 analysis adjusted for increased case and hospitalization incidence found that among 16–24-year-old males, every million BNT162b2 vaccine doses administered was expected to result in 112 myocarditis cases but also prevent 4690 hospitalizations over a 1-year period; 400 intensive care unit (ICU) admissions and 6 deaths would also be avoided during the first 120 days [75]. For 25–29-year-old males, estimates included fewer myocarditis cases and a greater number of hospitalizations, ICU admissions, and deaths averted. Potential benefits of vaccination against MIS-C and prolonged symptoms were not considered, which is particularly important given a CDC analysis (March 2020–January 2021; n = 36,005,294 patients) identifying an average 15.7-fold higher myocarditis risk among patients with versus without COVID-19 [76]. Importantly, VAERS reports can be made by anyone; they are therefore often incomplete and lack evidence regarding vaccine-related causality of reported events [77]. 

Outside of the United States, 1 and 12 cases of myocarditis were reported among Israeli 12–15-year-olds ≤21 days after dose 1 (n = 404,407) and ≤1 week after dose 2 (n = 326,463), respectively, during June–October 2021; 12 of 13 cases occurred in males [78]. Respective risk estimates ≤21 days after each dose were 0.56 and 8.09 per 100,000 among males. Additionally, in Israel, a large, real-world study using data from matched vaccinated and unvaccinated persons (n = 884,828 each), as well as matched ≥16-year-olds with and without SARS-CoV-2 infection (n = 173,106 each), identified much higher risk ratios of myocarditis and pericarditis following SARS-CoV-2 infection compared with mRNA vaccination [26]. A UK study using national data (December 2020–August 2021; n = 38,615,491) estimated 1–10 extra myocarditis events per million individuals in the month postvaccination compared with 40 per million associated with SARS-CoV-2 infection [79]. 

Recent studies indicate that most cases of suspected COVID-19 vaccine myocarditis occurring in <21-year-olds (n = 139; United States and Canada) or 12–15-year-olds (n = 13; Israel) have a mild clinical course with rapid symptom resolution [78,80]. Among 1195 adjudicated and confirmed myocarditis cases in <30-year-olds following mRNA COVID-19 vaccination reported to VAERS as of September 30, 2021, 96% (784/813) of cases were hospitalized, with 98% (747/762) of those having been discharged by the time of the review; 87% (577/661) of discharged individuals had recovered, and no deaths were reported [81]. The risk of myocarditis was highest after the second mRNA vaccine dose in adolescent males and young men. Importantly, a recent analysis identified more rapid cardiac function recovery associated with mRNA-vaccine-related myocarditis (n = 9) compared with classic (n = 43) or MIS-C-associated myocarditis (n = 149) [82]. Several post-marketing studies are planned to evaluate myocarditis/pericarditis following BNT162b2 receipt [54].

### 3.3. Vaccination Recommendations

Public health organizations differ regarding recommendations for pediatric COVID-19 vaccination (Figure 1), partly because of the very early stage of vaccination programs globally and limited vaccine supply for this age cohort (Table 2). The US CDC recommends the vaccination of all 5–17-year-olds with either a two-dose BNT162b2 or mRNA-1273 primary series and all 6-month–4-year-olds with either a three-dose BNT162b2 or two-dose mRNA-1273 primary series (Figure 1) [83]. A BNT162b2 booster (third dose) is recommended for ≥5-year-olds. For moderately or severely immunocompromised individuals, three, four, and five total BNT162b2 doses are recommended for 6-month–4-year-olds, 5–11-year-olds, and 12–17-year-olds, respectively. For mRNA-1273, three total doses are recommended for those 6-month–17-year-olds who are moderately or severely immunocompromised. 

The UK Joint Committee on Vaccination and Immunisation (JCVI) recommends a two-dose COVID-19 series for 12–17-year-olds, with a longer interval (≥12 weeks for healthy individuals) between doses based on potential associations with lower myocarditis reporting rates and short-term immunogenicity and effectiveness [84,85]. Booster doses are recommended for all 16–17-year-olds and 12–15-year-olds who are in or have household members in specified risk groups [86]; a third dose is recommended for individuals who are in or have household members in specified risk groups (e.g., immunocompromised) [87]. JCVI currently recommends vaccination of 5–11-year-olds who are in a clinical risk group or are household contacts of someone who is immunocompromised [86]. For all children and adolescents, BNT162b2 is the vaccine of choice [84,85,86]. As previously noted, early real-world vaccine effectiveness assessments among adolescents support robust BNT162b2 effectiveness following a second dose given 8–12 weeks after dose 1 [71]. 

In a November 2021 statement, the WHO urged countries to evaluate their individual epidemiologic and social situations when considering COVID-19 pediatric vaccination, stressing that comparatively milder disease observed in children necessitates prioritizing vaccination of adults and at-risk groups; indirect health benefits of vaccination, including reduced transmission and minimizing educational and social disruptions, should also be considered [14]. Importantly, this statement emphasized that vaccination benefits outweighed risks, including myocarditis.

### 3.4. Parental Vaccine Hesitancy

Parental vaccine hesitancy toward COVID-19 is an evolving issue during the current phase of immunization against SARS-CoV-2. In a large UK study (n = 33,556), children and adolescents aged 9–18 years expressed a strong desire to be vaccinated, with 50.1% of participants indicating that they were “eager” or “willing” to have a COVID-19 vaccine if it was offered and 37.0% were undecided (i.e., “not bothered” or “didn’t know”) [88]. Responses differed by age, with a larger proportion of the older age groups being certain of opting in to the vaccine (approximately 75% of adolescents aged 16–18 years) and fewer undecided (<20%) [88].

However, the majority of data regarding vaccine hesitancy in the pediatric population is largely driven by parent opinion [88]. A national online US survey reported vaccine hesitancy in 28.9% of 637 parents of adolescents aged 12–15 years before COVID-19 vaccines being approved in this age group [89]. Compared with vaccine-accepting parents, vaccine-hesitant parents were less informed about vaccines, more accepting of vaccine conspiracies, and less concerned regarding health risks of COVID-19 in children [89]. Primary concerns of vaccine-hesitant parents involved safety rather than vaccine effectiveness [89]. Similar studies in other countries report high levels of COVID-19 vaccine hesitancy among parents of children or adolescents, with safety of the vaccines being a common reason for concern [90,91,92,93].

While there are ethical considerations for the vaccination of children and adolescents against their parents’ wishes, some countries permitted an exception to the requirement of parental consent specifically in the case of COVID-19 vaccines, deeming parent refusal as being not in the best interests of the child and potentially harmful based on medical recommendations [94]. Indeed, the Australian government indemnified clinicians administering COVID-19 vaccines to adolescents aged 12–17 years [94].

**Table 2:** A sampling of recommendations by public health organizations regarding COVID-19 vaccination in children.

	CDC [83]			JCVI [84,85,86,87,95]		WHO [14]	
	Children 6 Months–4 Years Old	Children 5–11 Years Old	Adolescents 12–17 Years Old	Children 5–11 Years Old	Adolescents 12–17 Years Old	Children 5–11 Years Old	Adolescents 12–17 Years Old
**Primary vaccination**	Recommended for all individuals Dosing: three 3 µg BNT162b2 doses (≥3 weeks between doses 1 and 2 and ≥8 weeks between doses 2 and 3); two 25 µg mRNA-1273 doses (≥4–8 weeks between doses)	Recommended for all individuals Dosing: two 10 µg BNT162b2 doses ≥3–8 weeks apart for those 5–11 years old; two 25 µg mRNA-1273 doses ≥4 weeks apart for those 5 years old; and two 50 µg mRNA-1273 doses ≥4–8 weeks apart for those 6–11 years old	Recommended for all individuals Dosing: two 30 µg BNT162b2 doses ≥3–8 weeks apart; two 100 µg mRNA-1273 doses ≥4–8 weeks apart	Should be offered to those in a clinical risk group or household contacts of immunosuppressed individuals Dosing: two 10 µg BNT162b2 doses 8 weeks apart	Should be offered to all individuals Dosing: two 30 µg BNT162b2 doses ≥8 (at-risk individuals) or ≥12 weeks (healthy individuals) apart	Countries should consider their specific epidemiologic and social context when considering vaccination of children and adolescents, with priority given to vaccination of adults and at-risk groups	
**Additional dose**	No recommendations given	Recommended for moderately and severely immunocompromised individuals Timing: ≥28 days after second dose		Not recommended	Should be offered to those who were severely immunosuppressed at the time of their first or second dose Timing: generally ≥8 weeks after second dose	No recommendations given	No recommendations given
**Booster dose**	Not recommended	Recommended (for BNT162b2 only) Timing: ≥5 months after last primary dose	Recommended (for BNT162b2 only) Timing: ≥5 months after last primary dose	Not recommended	12–15 years of age: should be offered to those in a clinical risk group, household contact of immunosuppressed individuals, or severely immunocompromised and received a third dose 16–17 years of age: should be offered to all individuals Timing: ≥3 months after last primary dose	No recommendations given	No recommendations given

CDC, US Centers for Disease Control and Prevention; COVID-19, coronavirus disease 2019; JCVI, UK Joint Committee on Vaccination and Immunisation; WHO, World Health Organization.

## 4. Conclusions

Despite the inclusion of children and adolescents in vaccine recommendations and ever-growing clinical data supporting pediatric vaccination, vaccination rates, including among older adolescents, have not been high [12,13]. This may reflect the later availability, limited supply, and lower prioritization of COVID-19 vaccination among pediatric compared with adult populations, questions about the long-term safety profile of novel mRNA vaccines in children, and vaccine hesitancy.

## Figures and Tables

**Table 1:** COVID-19 vaccine clinical trials in children and adolescents.

Vaccine (Type)	Characteristic	Details
**Infants, children, and adolescents**		
**CoronaVac** **(inactivated vaccine)** [49]	ClinicalTrials.gov identifier	NCT04551547
	Phase (design)	1/2 (randomized, controlled)
	Age group	3–17 years
	Dose (schedule)	1.5 or 3.0 µg (2 doses 28 days apart)
	Number of participants	72 (phase 1) and 480 (phase 2)
	Immunogenicity	Humoral responses were induced, with neutralizing antibody titers induced by the 3.0 µg dose higher than those of the 1.5 µg dose (the data support the 3.0 µg dose for further development in this age group)
	Safety	The most common reactions were injection site pain and fever; most were mild or moderate and transient
**CoronaVac** **(inactivated vaccine)**	ClinicalTrials.gov identifier	NCT04992260
	Phase (design)	3 (randomized, placebo-controlled)
	Age group	6 months–17 years
	Number of participants	~14,000
	Endpoints	Efficacy of RT-PCR–confirmed symptomatic COVID-19 (primary), safety, immunogenicity
	Primary completion date	May 2022 (estimated)
**BBIBP-CorV** **(inactivated vaccine)** [50]	Clinical trial identifier	ChiCTR2000032459
	Phase (design)	1/2 (randomized, controlled)
	Age group	3–17 years
	Dose (schedule)	2, 4, or 8 µg (3 doses on days 0, 28, and 56)
	Number of participants	288 (phase 1) and 720 (phase 2)
	Immunogenicity	All doses elicited robust humoral responses; the 4 µg dose on a 2-dose regimen (21 days apart) will be further studied in this age group
	Safety	The most common reactions were injection site pain and fever; most were mild or moderate
**Adolescents**		
**BNT162b2** **(mRNA vaccine)** [46]	ClinicalTrials.gov identifier	NCT04368728
	Phase (design)	3 (randomized, placebo-controlled)
	Age group	12–15 years
	Dose (schedule)	30 µg (2 doses 21 days apart)
	Number of participants	2260
	Follow-up	At the time of the report, 58% of participants had ≥2 months of follow-up after dose 2
	Vaccine efficacy	Observed vaccine efficacy of 100% (95% CI: 75.3, 100)
	Immunogenicity	Immune response was noninferior to that observed in 16–25-year-olds
	Safety	Injection site pain was the most common local reaction, and headache and fatigue were the most common systemic events; these events were mostly mild to moderate in severity and transient
**mRNA-1273** **(mRNA vaccine)** [48]	ClinicalTrials.gov identifier	NCT04649151
	Phase (design)	2/3 (randomized, placebo-controlled)
	Age group	12–17 years
	Dose (schedule)	100 µg (2 doses 28 days apart)
	Number of participants	3726
	Follow-up	83 days
	Vaccine efficacy	Using CDC definition of COVID-19 with onset of 14 days after dose 2: 93% (95% CI: 47.9, 99.9) In the per-protocol population with an onset of 14 days after dose 2: 56% (95% CI: 16.8, 76.4)
	Immunogenicity	Immune response was noninferior to that observed in 18–25-year-olds
	Safety	Injection site pain was the most common local reaction, and fatigue and headache were the most frequently reported systemic events; these were most commonly grade 1/2 and transient
**Children**		
**BNT162b2** **(mRNA vaccine)** [47]	ClinicalTrials.gov identifier	NCT04816643
	Phase (design)	2/3 (randomized, placebo-controlled)
	Age group	5–11 years
	Dose (schedule)	10 µg (2 doses 21 days apart)
	Number of participants	2268
	Follow-up	Median of 2.3 months (range 0–2.5 months)
	Vaccine efficacy	Observed vaccine efficacy of 90.7% (95% CI: 67.7, 98.3)
	Immunogenicity	Immune response was noninferior to that observed in 16–25-year-olds
	Safety	Injection-site pain was the most common local reaction, and fatigue and headache were the most frequently reported systemic events; these events were mostly mild to moderate in severity and transient
**mRNA-1273** **(mRNA vaccine)** [64]	ClinicalTrials.gov identifier	NCT04796896
	Phase (design)	2/3 (randomized, placebo-controlled)
	Age group	6–11 years
	Dose (schedule)	50 µg (2 doses 28 days apart)
	Number of participants	4016
	Follow-up	Median 82 days after dose 2
	Vaccine efficacy	Using CDC definition of COVID-19 with onset of 14 days after dose 2: 88% (95% CI: 70.0, 95.8)
	Immunogenicity	Immune response was noninferior to that observed in 18–25-year-olds who received 2 doses at the 100 µg dose level
	Safety	The most common local adverse reaction was injection site pain, and the most common systemic adverse reactions were headache and fatigue
**Infants**		
**BNT162b2** **(mRNA vaccine)** [57]	ClinicalTrials.gov identifier	NCT04816643
	Phase (design)	2/3 (randomized, placebo-controlled)
	Age group	6 months–4 years
	Dose (schedule)	Dose 1 and 2 administered 3 weeks apart; dose 3 administered ≥8 weeks after dose 2
	Number of participants	1776 (6–23 months of age); 2750 (2–4 years of age)
	Follow-up	Median 1.3–1.4 months after dose 3
	Vaccine efficacy	N/A
	Immunogenicity	Immune response after 3 doses was noninferior to that observed after 2 doses in 16–25-year-olds
	Safety	In participants 6–23 months of age, adverse reactions after any dose included irritability (68%), decreased appetite (39%), injection site tenderness (26%), injection site redness (18%), fever (14%), injection site swelling (7%), and lymphadenopathy (0.2%). In participants 2–4 years of age, these included injection site pain (47%), fatigue (45%), injection site redness (19%), fever (11%), headache (9%), injection site swelling (8%), chills (6%), muscle pain (5%), joint pain (2%), and lymphadenopathy (0.1%).
**mRNA-1273** **(mRNA vaccine)** [61]	ClinicalTrials.gov identifier	NCT04796896
	Phase (design)	2/3 (randomized, placebo-controlled)
	Age group	6 months–5 years
	Dose (schedule)	25 µg (2 doses 1 month apart)
	Number of participants	6388
	Follow-up	Median of 68–71 days after dose 2
	Immunogenicity	Immune response was noninferior to that observed in 18–25-year-olds who received 2 doses at the 100 µg dose level
	Safety	The most common adverse reactions in those 6–23 months of age were irritability/crying (64–68%), pain (37–46%), sleepiness (35–37%), and loss of appetite (30–32%). In those 24–36 months of age, these were pain (53–68%), irritability/crying (54–55%), and sleepiness (24–31%). In those 37 months–5 years of age, these were pain (65–73%) and fatigue (40–48%).

CDC, US Centers for Disease Control and Prevention; RT-PCR, reverse transcription polymerase chain reaction.

## Data Availability

Not applicable.

## Supplementary Materials

The following supporting information can be downloaded at: https://www.mdpi.com/article/10.3390/vaccines10122039/s1. Figure S1. Comparison of Immune Response to SARS-CoV-2 Infection in Pediatric and Adult Patients With COVID-19 [35].
