# Supplementary material for: COVID-19 Epidemiology, Immunity, and Vaccine Development in Children: A Review
Source: Vaccines (Basel). 2022 Nov 29;10(12):2039. doi: 10.3390/vaccines10122039 (PMC9781884; doi:10.3390/vaccines10122039)
Supplement: Supplementary file 1 [file vaccines-10-02039-s001.zip › vaccines-1960256-supplementary.docx]

# Supplementary Materials

**Figure S1.** Comparison of Immune Response to SARS-CoV-2 Infection in Pediatric and Adult Patients With COVID-19 [35]


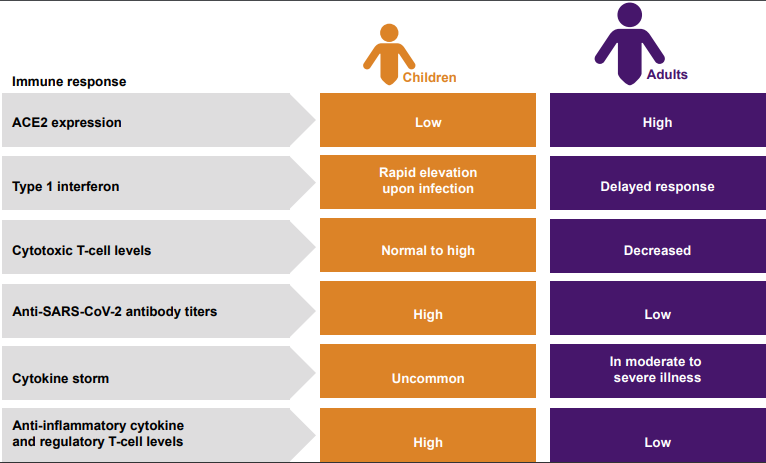
 ACE2, angiotensin-converting enzyme 2; COVID-19, coronavirus disease 2019; SARS-CoV-2, severe acute respiratory syndrome coronavirus 2.
